# Waterborne Outbreak of Norwalk-Like Virus Gastroenteritis at a Tourist Resort, Italy

**DOI:** 10.3201/eid0806.010371

**Published:** 2002-06

**Authors:** Della Boccia, Alberto Eugenio Tozzi, Benvon Cotter, Caterina Rizzo, Teresa Russo, Gabriele Buttinelli, Alfredo Caprioli, Maria Luisa Marziano, Franco Maria Ruggeri

**Affiliations:** * Istituto Superiore di Sanità, Rome, Italy; † University of Bari, Bari, Italy; ‡ Local Health Unit, Montalbano Jonico, Matera, Italy; § European Programme for Intervention Epidemiology Training (EPIET), Rome, Italy

**Keywords:** gastroenteritis, Norwalk-like virus, Italy, outbreak, water

## Abstract

In July 2000, an outbreak of gastroenteritis occurred at a tourist resort in the Gulf of Taranto in southern Italy. Illness in 344 people, 69 of whom were staff members, met the case definition. Norwalk-like virus (NLV) was found in 22 of 28 stool specimens tested. The source of illness was likely contaminated drinking water, as environmental inspection identified a breakdown in the resort water system and tap water samples were contaminated with fecal bacteria. Attack rates were increased (51.4%) in staff members involved in water sports. Relative risks were significant only for exposure to beach showers and consuming drinks with ice. Although Italy has no surveillance system for nonbacterial gastroenteritis, no outbreak caused by NLV has been described previously in the country.

*Norwalk virus* is the prototype of the genus Norwalk-like virus (NLV) in the *Caliciviridae* family, which includes a large number of genetically related strains that together represent the most important cause of gastroenteritis outbreaks worldwide (*1*,*2*). NLV accounts for up to 96% of outbreaks of nonbacterial gastroenteritis in the United States (*3*) and has been implicated in 43% of all foodborne outbreaks in England, 67% in Sweden, and 80% in the Netherlands (*4*–*6*).

Outbreaks of NLV gastroenteritis more frequently affect adults and children >5 years of age. Because of the low infectious dose of the agent (10–100 viral particles can induce symptoms), outbreaks are characterized by a high secondary attack rate (*7*). In most documented outbreaks, the incubation period has been reported as 24–48 hours; the average duration of symptoms is 12–60 hours. During an outbreak, >50% of infected persons have symptoms of vomiting, most often in combination with diarrhea (*8*). The main source of infection is usually contaminated food or water (*9*–*13*), while the usual mode of transmission is direct person-to-person contact with saliva, vomit, or aerosols. Transmission may also occur through contact with contaminated objects and surfaces such as showers, sinks, mats, and floors (*3*).

In Italy, which has no surveillance system for nonbacterial gastroenteritis, the impact of NLV infection is unknown, and no previous outbreaks of confirmed NLV infection have been reported. We describe a large outbreak of gastroenteritis caused by NLV at a resort in Italy.

## Methods

**Figure 1:**
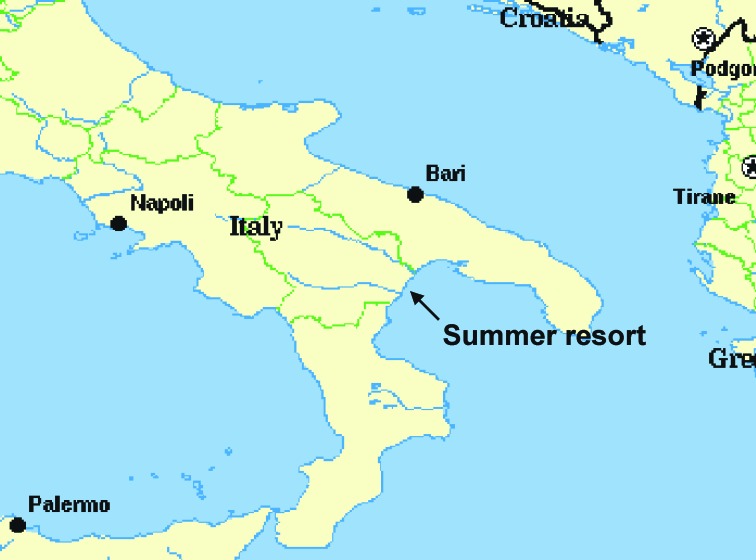
Map of Italy, showing location of tourist resort on Gulf of Taranto.

The outbreak occurred at a tourist resort in the Gulf of Taranto, southern Italy, during July 7–31, 2000 (Figure 1). The resort has an area of 122 hectares with 456 guest rooms in 19 buildings, in addition to staff quarters. The buildings are situated around a central area where a restaurant, a swimming pool, and the resort management office are located. The resort can accommodate 1,000 guests, who usually arrive on a Saturday and depart 1 or 2 weeks later, resulting in approximately 50% turnover of guests each weekend.

**Figure 2:**
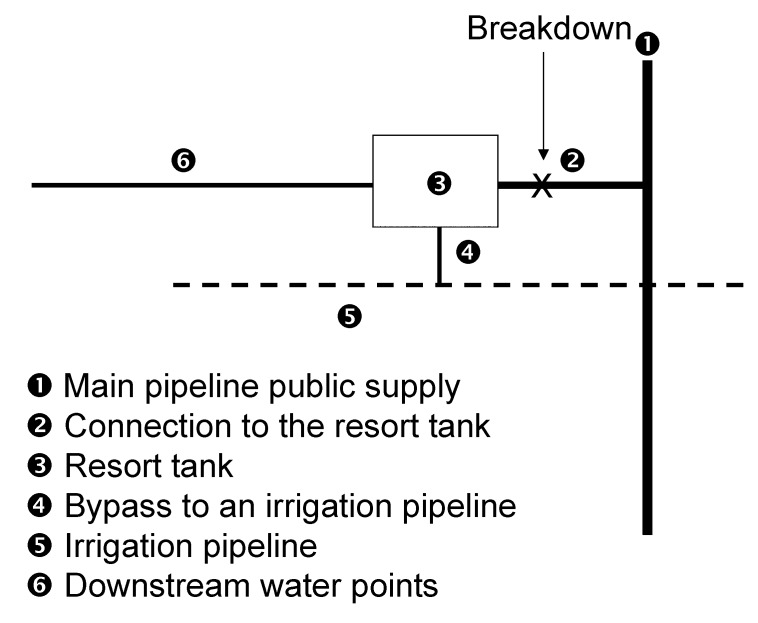
Water supply system in tourist resort, Italy.

The resort’s water tank is supplied via a 1-km pipe connected to the main public water supply (Figure 2). On July 13, a break in this water pipe was observed (Figure 2, point 2). Inspection also showed a bypass connecting the tank to an unused irrigation system (Figure 2, points 4 and 5).

On July 18, 2000, the local health unit and the Institute of Hygiene of the Faculty of Medicine in Bari were notified about an outbreak of gastroenteritis at the resort. An epidemiologic investigation was initiated the same day to identify the agent and the mode and vehicle of transmission and to implement control measures. By July 20, when the local health unit notified the Istituto Superiore di Sanità in Rome, the outbreak had already been in progress for approximately 2 weeks and >150 persons were ill.

### Outbreak Investigation

A case was defined as illness in any guest or employee who stayed at the tourist resort during the period July 1–31 and who had diarrhea (three or more loose stools in any 24-hour period) or vomiting (at least one episode) or both, in the same period. Case finding was done by checking records of the resort medical center; after July 20, a door-to-door search was initiated. Demographic data and information on symptoms were collected in face-to-face interviews by the medical staff of the local health unit and the University of Bari.

Because of the high number of cases in resort staff members, a retrospective cohort study was performed to assess risk factors associated with illness in this group. Persons eligible for the study were staff members employed at the resort from July 1 to 31. Standard questionnaires were sent to all 224 staff members in the first week of August. Information requested included name, date of birth, sex, room number, job type, date of onset and type of symptoms, and water and food preferences. A month had elapsed between onset of symptoms and distribution of the questionnaires. We did not inquire about actual food history and activities of staff members during the outbreak but rather about their food preferences and usual activities.

### Statistical Analysis

The questionnaires from guests and staff members were returned to the Istituto Superiore di Sanità, where the data were analyzed by using SPSS Base 10.0 (SPSS Inc., Chicago, IL) and Epi-Info 6.04 (Centers for Disease Control and Prevention, Atlanta, GA). Information collected on cases was used to construct the epidemic curve and describe the clinical presentation of the disease. Attack rates, denominator data, personal characteristics, and clinical symptoms of cases were compared between guests and staff members by chi square or Fisher exact test when appropriate; the Mann-Whitney U-test was used for comparisons of age. The room location of ill persons was plotted on a map of the resort that included water pipelines in an attempt to identify any clustering of cases along the pipeline. Statistical test for clustering was performed by the cluster k-means method with SPSS Base 10.0 (SPSS Inc.).

In the cohort study, the attack rate was calculated for the total staff and also by specific job type. Relative risks and 95% confidence intervals were also calculated for job type, behaviors and activities, and food preferences.

### Laboratory Investigations

From July 18 to 28, samples (28 fecal and 2 vomit specimens) were collected from 30 participants whose illness met the case definition. Part of each specimen was stored at -20°C until examination for viral particles and free fecal cytotoxins, and the rest was refrigerated and processed within 12 hours of collection.

Ova and parasites were detected by direct microscopy, and *Salmonella, Shigella, Campylobacter*, *Yersinia enterocolitica, Staphylococcus aureus*, and enteropathogenic *E. coli* were sought by standard methods (*14*). The presence of *Clostridium perfringens* enterotoxin (CPE) was determined either by assaying the cytopathic effect on Vero cells or by reverse passive latex agglutination (RPLA) test (Oxoid Italia Spa, Garbagnate Milanese, Milan) according to the manufacturer’s instructions (*14*).

Stool and vomit suspensions were examined by NLV-specific reverse transcription/polymerase chain reaction (RT/PCR) with generic primers JV12–JV13 to a consensus sequence on the RNA polymerase segment of the genome shared by most NLV strains (*15*). For confirmation of the diagnosis, gels were further analyzed by Southern blot with a mixture of NLV-specific probes (*15*). The 327-bp amplification product was subjected to sequence analysis with PCR primers, and the sequences obtained were aligned with those in the European Molecular Biology Laboratory Nucleotide Data Bank.

After July 13, water samples were repeatedly collected from the main public water supply and from various points outside and inside the resort. Samples from food in the kitchen and the refrigerators were collected and sent to the University of Bari on July 18. Water and food samples were subjected to culture tests for enteric bacterial pathogens, according to standard methods.

## Results

### Descriptive Epidemiology

Of 344 cases identified from July 1 to 31, 69 (20%) were in staff members. Information on personal characteristics and clinical presentation was available for 248 ill persons (Table 1). Diarrhea, vomiting, and abdominal pain were observed in >70% of all cases. Five patients were hospitalized; all recovered rapidly and were discharged within a few hours. None of the patients had any further sequelae. Attack rates did not differ by age, sex, or symptoms for cases in guests or staff members. For cases in guests, the median interval from the arrival date at the resort and onset of symptoms was 4 days, and symptoms developed in 77% within 5 days.

**Table 1:** Personal characteristics of ill persons and clinical symptoms, tourist resort, Italy, July 2000

Characteristics	Guests	Staff members	Total
Information available (n)	179	69	248
Age (years); mean (range)	23 (1–88)	26 (19–53)	24 (1–88)
Female (%)	52	54	53
Diarrhea (%)	93	92	93
Vomiting (%)	84	72	80
Fever (%)	53	58	54
Abdominal pain (%)	63	83	70
Hospitalization (%)	2.2	1.4	2.0

**Figure 3:**
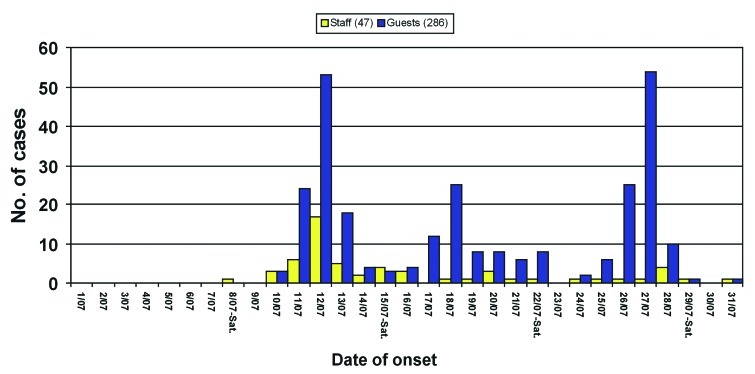
Cases of gastroenteritis with known date of onset (n=333) in guests and staff members at a tourist resort, Italy, July 2000.

The epidemic curve shows three distinct peaks in each of the 3 weeks, beginning on July 12 (70 cases), July 18 (26 cases), and July 27 (55 cases). Over the total outbreak period, 275 cases occurred in guests and 58 in staff (Figure 3). Fifty-seven percent of cases in staff members occurred before July 15. The outbreak lasted 24 days, and no cases were observed after July 31.

Because of the rapid turnover at the resort, attack rates for guests were calculated separately for each week: an attack rate of 102 (10.5%) of 970 was observed in week 1; 66 (8.7%) of 760 in week 2; and 105 (10.1%) of 1,034 in week 3. Ill guests occupied 157 of the resort’s 456 rooms. No significant evidence of either clustering by the cluster k-means methods (p=0.392) or increased frequency of cases in rooms near the water pipeline was observed. Attack rates by sex, age group, and week of stay were similar.

### Analytical Epidemiology

For the analysis of risk factors in the cohort study, 181 questionnaires from 224 staff members were completed and analyzed. The attack rate in this group was 69 (38.1%) of 181. The lowest attack rates were observed in staff members who worked in the kitchen or the office, and the highest were in waiters, sports trainers, entertainers, and cleaning staff (i.e., staff members who are have close contact with guests) (Table 2). Staff members who took showers on the beach or consumed drinks with ice were more likely to become ill than those who did not. No association was found between disease and eating any particular type of food or with being at work on July 8–11 (the first days of the outbreak) (Table 3).

**Table 2:** Attack rates and relative risks for staff members (n=69) according to type of work, tourist resort, Italy, July 2000

Type of work	Attack rate (%)	Relative risk	95% CI ^a^
Kitchen staff	4/34 (11.8)	Referent	
Office staff	3/14 (21.4)	1.8	0.5–7.1
Bar staff	3/11 (27.3)	2.3	0.6–8.8
Shop assistant	7/21 (33.3)	2.8	0.9–8.5
Cleaning staff	16/36 (44.4)	3.8	1.4–10.2
Sports trainers and entertainers	19/37 (51.4)	4.4	1.6–11.5
Waiters	17/28 (60.7)	5.2	2.0–13.6

^a^CI, confidence interval.

**Table 3:** Attack rates and relative risks according to usual behaviors and activities of staff members, tourist resort, Italy, July 2000

Exposure	No. (n=69)	No. exposed	Attack rate (%)	Relative risk	95% CI ^a^
Shower on the beach	22	14	63.6	1.8	1.2–2.6
Swimming in the pool	45	22	48.9	1.4	0.9–2.0
Drinking tap water	104	47	45.2	1.4	0.9–2.2
Drinks with ice	128	55	43.0	1.8	1.0–3.2
Swimming in the sea	72	31	43.0	1.2	0.8–1.7
Eating at resort restaurant	159	64	40.2	1.5	0.5–3.9
Eating ice cream	140	56	40.0	1.1	0.6–1.9
Eating meat	151	60	39.7	1.2	0.6–2.4
Eating salad	123	48	39.0	1.0	0.6–1.6
Eating fruit	139	54	38.8	1.0	0.6–1.8
Eating pasta	142	55	38.7	1.2	0.6–2.1
Consuming drinks on draught	91	35	38.5	1.0	0.7–1.4
Eating fish	112	40	35.7	0.7	0.5–1.1
Eating seafood	85	28	32.9	0.7	0.5–1.1

^a^CI, confidence interval.

### Microbiologic Results

Stool samples from 28 patients were negative for ova and parasites and bacterial enteropathogens. Of the 28 stool samples examined by NLV-specific RT-PCR, 22 had an amplified DNA of the size expected for NLV. The 327-bp amplification product was also confirmed for all samples by Southern blot hybridization with NLV-specific probes. Vomit specimens from two other subjects were negative.

A readable common sequence of 290 bp was obtained with sequence analysis and found to be the same for eight samples, indicating a single outbreak virus strain. The sequence was analyzed against the European Molecular Biology Laboratory Nucleotide Data Bank, yielding a best fit with the RNA polymerase sequence of the Lordsdale strain of NLV (*16*). Nucleotide identity between the two strains was 93.1% (270/290 residues), indicating that the outbreak NLV strain belongs to GGII.

When the stool supernatants stored at -20°C were examined by the Vero cell assay for free bacterial toxins, a CPE consistent with that of *C. perfringens* enterotoxin was induced by seven samples. The RPLA test confirmed the presence of *C. perfringens* enterotoxin in all seven samples. The positive specimens had been collected July 18–21, and all were also positive for NLV (Table 4).

**Table 4:** Presence of Norwalk-like virus and *Clostridium perfringens* enterotoxin in stool samples from ill persons, Italy, July 2000

Sampling date	No. examined	No. positive for NLV ^a^	No. positive for CPE	No. positive for NLV + CPE
07/18/01	12	9	0	0
07/20/01	1	1	1	1
07/21/01	8	7	6	6
07/27/01	2	2	0	0
07/28/01	5	3	0	0
Total	28	22	7	7

^a^Abbreviations used: NLV, Norwalk-like virus; CPE, *Clostridium perfringens* enterotoxin.

All food samples tested were negative for enteropathogenic bacteria. Water samples collected on July 13 from faucets in the bar, the kitchen, and a guest room (Figure 2, point 6) had high levels of coliforms (up to 130 CFU/mL) and fecal streptococci (up to 22 CFU/mL). The same level of contamination was observed in water samples from the pipe connecting the resort to the public water supply (Figure 2, point 2); samples collected from the public water supply outside the resort (Figure 2, point 1) were always negative. After July 15, when chlorine was added to the tank, the level of contamination of tap water inside the resort steadily decreased; no contamination was detectable after superchlorination on July 22.

## Discussion

Although NLV gastroenteritis epidemics likely occur as frequently in Italy as in the rest of Europe, to our knowledge this is the first outbreak of NLV infection to be confirmed in the country. It affected many guests and employees at a summer vacation resort and involved high attack rates in all age groups. The actual number of cases has likely been underestimated since persons with a mild illness may not have sought medical attention. In fact, the retrospective investigation of staff members showed an attack rate three times higher than in guests.

This outbreak had an unusual pattern, with three regular peaks occurring at constant intervals for 3 weeks. This pattern, which is compatible with a point-source infection (*3*), may be explained by the rapid turnover of guests and their periodic replacement with susceptible persons in the presence of a constant exposure to infection. Most guests arrived at the resort on a Saturday and stayed 1–2 weeks. Guests who became ill did so a few days after their arrival, suggesting that exposure to a source of infection was relatively constant during the whole period. Moreover, a large proportion of staff members had onset of illness in the first week of the outbreak. The hypothesis of a common source of infection is further supported by the identical nucleotide sequence detected in viruses from eight patients during the outbreak.

Water was the likely source of this outbreak. Environmental inspection identified a breakdown in the water system of the resort, and tap water samples from different places in the resort showed contamination with fecal bacteria. Although microbiologic testing for NLV could not be performed on drinking or recreational water, the presence of fecal bacteria suggests that the water system may have been the actual source of NLV. Despite the possible passage of the virus through several hosts during the outbreak, the genome segment used for diagnosis showed complete stability, suggesting that a very high number of human passages may be required to produce the known nucleotide variability for NLV, at least in the RNA polymerase region.

Some specimens showed evidence of simultaneous infection with NLV and enterotoxigenic *C. perfringens.* The food item(s) that could have been the source of infection by *C. perfringens* remained unknown. However, the presence of *C. perfringens* enterotoxin in a small, defined cluster of patients (7 of 28 stool samples) and the concomitant presence of NLV in the 7 positive stools suggests that *C. perfringens* played only a minor role, if any, in the outbreak.

Control measures to limit the spread of the infection had no effect, probably because they did not address the point source and failed to prevent person-to-person transmission. After July 15, 2000, the consumption of tap water was banned, and only bottled mineral water was served in the resort restaurant and used to wash vegetables. Water from the main tank, however, continued to be used for showers, to make ice for consumption (through July 28), and for irrigation. Furthermore, on July 22, the bypass pipe was removed, the water inside the resort tank underwent superchlorination, and the pipe connecting the resort to the public water supply was shut down. However, NLV do survive high levels of chlorination (*3*,*8*), and the treatment was performed only once, at a late stage of the outbreak, and at only one point upstream of the resort water system. Finally, although the resort was serviced by a mobile tank truck that provided water from the main public water supply, the resort main tank was never emptied and cleaned before treatment. Therefore, after July 22, contaminated residual water could have been gradually diluted by refilling the resort tank with clean water from mobile tanks.

In the cohort study of staff members, having showers on the beach was identified as a risk factor, while consuming drinks with ice was only weakly associated with illness. No exposure to other water sources, including drinking tap water (the use of which was forbidden after July 15) was significant. Our analysis found no evidence that contaminated food was the source of infection: no food preference was associated with an increased risk of being ill, and personnel working in the kitchen had the lowest attack rate.

In addition to water contamination, person-to-person transmission may have played a role in this outbreak. Typically staff members of tourist resorts share the same living quarters and have frequent contact with guests during meals, sport training, entertainment, and other activities. Person-to-person transmission may also explain the fact that the time between arrival and onset of symptoms in guests was longer than the incubation period expected for NLV. Person-to-person transmission of NLV infection is well documented (*8*), and secondary cases may occur. Airborne and fomite transmission also may facilitate the spread of the virus during outbreaks. Such hypothesis is confirmed by the higher attack rate in the cleaning staff and in staff members working in close contact with guests.

This investigation had several limitations. Since the cohort study was carried out after the outbreak had ended, we could inquire only about food preferences and usual activities rather than actual food histories and activities before the outbreak. Recall bias may have occurred, which may have led to nondifferential misclassification of exposure and underestimation of the observed relative risks. If NLV had spread through water and person-to-person transmission had occurred, virtually everyone in the resort would have been exposed to the agent and any epidemiologic association would be difficult to find. Finally, no test specific for NLV was performed on water samples, and the hypothesis of water as the actual source of infection cannot be confirmed.

In conclusion, this event confirms that large outbreaks due to NLV may be occurring in Italy, but without the use of appropriate diagnostic methods this pathogen may go unrecognized. This occurrence highlights the need for a surveillance system of such outbreaks in cooperation with laboratories capable of diagnosing viral gastrointestinal infections.
